# Supplementary material for: Behavioral, Electrophysiological, and Toxicological Responses of Plutella xylostella to Extracts from Angelica pubescens
Source: Insects. 2023 Jul 6;14(7):613. doi: 10.3390/insects14070613 (PMC10380822; doi:10.3390/insects14070613)
Supplement: Supplementary file 1 [file insects-14-00613-s001.zip › insects-2381414-supplementary.pdf]

Supplementary materials

Table S1. List of tested compounds for *A. pubescens* extract

Reagent type(species)	CAS_ num	Class	Company	Purity
Artemisinin	63968-64-9	polyene-alkynes	Aladdin	98%
4-Methylbenzylidene camphor	36861-47-9	polyene-alkynes	Aladdin	≥98.0%
Santonin	481-06-1	polyene-alkynes	Macklin	98%
(-)-Caryophyllene oxide	1139-30-6	polyene-alkynes	Macklin	95%
Nootkatone	4674-50-4	polyene-alkynes	Macklin	98%
Ethyl caffeate	102-37-4	phenolic acids	Macklin	98%
2-Hydroxycinnamic acid	614-60-8	phenolic acids	Aladdin	97%
Caffeic acid	331-39-5	phenolic acids	Macklin	98%
3,4-Dimethoxycinnamic acid	2316-26-9	phenolic acids	Macklin	99%
4-Coumaric acid	7400-08-0	phenolic acids	Aladdin	97%

LC-MS analysis

LC-MS of the *A. pubescens* extract was performed by Sci-tech Innovation Quality Testing Company (Qingdao, China). Accurately weighed 100mg of *A. pubescens* extract and placed into a 2 mL centrifuge tube. We added 1mL of 70% methanol and 3mm steel balls, and broke the mixture for 3 min, followed by sonication at 40KHZ for 10 min at a lower temperature, followed by centrifugation at 11000 g for 10 min at 4°C. The supernatant was diluted 2-100, placed in standard internal solution 2-amino-3-(2-chloro-phenyl)-propionic acid, filtered through 0.22 μm PTFE.

A. pubescens extract compounds were analyzed by LC-MS system (Thermo, Ultimate 3000LC, and Q-Exactive HF, Thermo Fisher Scientific, Waltham, MA, USA). HPLC separation was carried out on a C18 analytical column (Zorbax Eclipse C18 (1.8μm*2.1*100mm) Agilent Technologies, Santa Clara, CA, USA), operated at 30°C. The mobile phase consisted of 0.1% (v/v) formic acid in water (eluent A) and acetonitrile (eluent B) (Tab. S2). The injection volume was 2 μL, and the autosampler temperature was 4°C.

Positive and negative ion mode: Sheath gas flow rate: 45arb; Sheath gas flow rate: 15arb; Purge gas flow rate: 1arb; Electron impact mass spectrometry was carried out at 3.5 kV; S-Lens RF Level: 55%. The source and capillary column temperatures were maintained at 325 and 330°C, respectively. Scan mode: the scanning mass range was from 100 to 1500 m/z, and data-dependent secondary mass spectrometry scanning was used; the resolutions were 120000 (MS) and 60000 (MS2). Peak finding, filtering, alignment and identification of compounds in the Thermo Mz Cloud and Thermo Mz Vault databases using Compound Discoverer 3.2 software.

Table S2. LC solvent system conditions

Time(min)	Flow rate (μL/min)	Gradient	B% acetonitrile
0-2	300	-	5
2-6	300	linearly	30
6-7	300	-	30
7-12	300	linearly	78
12-14	300	-	78
14-17	300	linearly	95
17-20	300	-	95
20-21	300	linearly	5
21-25	300	-	5

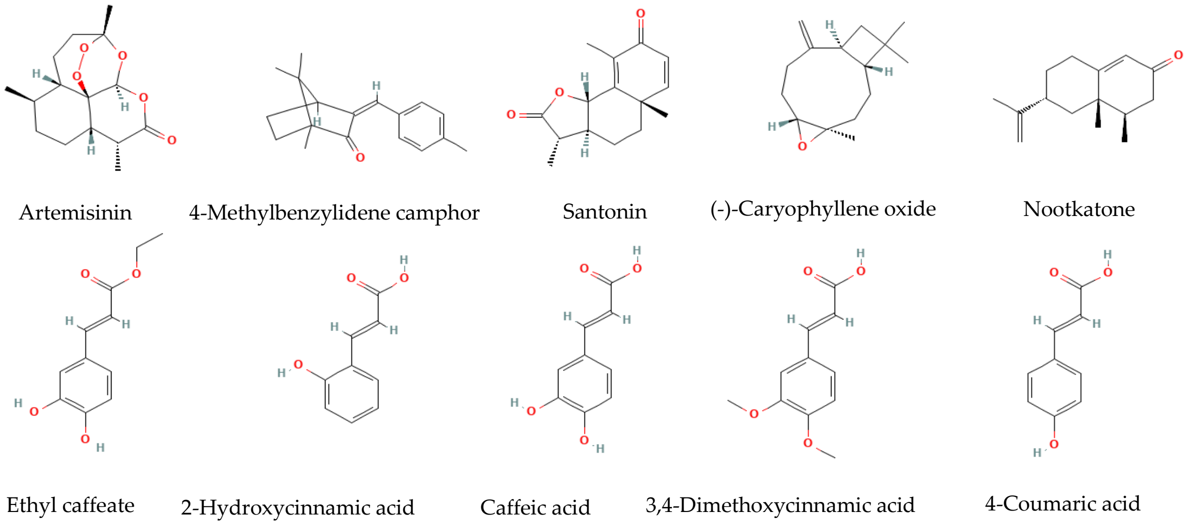

Figure S1. Structures of 10 chemical constituents isolated from *A. pubescens* extract
